# A consumer-driven bioeconomy in housing? Combining consumption style with students' perceptions of the use of wood in multi-storey buildings

**DOI:** 10.1007/s13280-020-01397-7

**Published:** 2020-10-09

**Authors:** Eliisa Kylkilahti, Sami Berghäll, Minna Autio, Jonne Nurminen, Ritva Toivonen, Katja Lähtinen, Heini Vihemäki, Florencia Franzini, Anne Toppinen

**Affiliations:** 1 Department of Economics and Management, University of Helsinki, Latokartanonkaari 5, P.O. Box 27, 00014 Helsinki, Finland; 2 Department of Forest Sciences, University of Helsinki, Latokartanonkaari 7, 00014 Helsinki, Finland; 3 Department of Educational Sciences, University of Helsinki, P.O. Box 8, 00014 Helsinki, Finland; 4 Natural Resources Institute Finland (Luke), Latokartanonkaari 9, 00790 Helsinki, Finland; 5 Helsinki Institute of Sustainability Science, Faculty of Agriculture and Forestry, University of Helsinki, Latokartanonkaari 7, 00014 Helsinki, Finland

**Keywords:** Consumer perception, Consumption styles, Sustainable bioeconomy, Urban housing, Wood, Young consumers

## Abstract

Consumer acceptance of new bio-based products plays a key role in the envisioned transition towards a forest-based bioeconomy. Multi-storey wooden buildings (MSWB) exemplify a modern, bio-based business opportunity for enacting low-carbon urban housing. However, there is limited knowledge about the differing perceptions consumers hold regarding wood as an urban building material. To fill this gap, this study explores Finnish students’ perceptions of MSWB relative to their familiarity with wooden residential buildings, and then connects these perceptions to ‘consumption styles.’ Data were collected in the Helsinki metropolitan area via an online questionnaire (*n* = 531). The results indicate that the aesthetic appearance of MSWB are appreciated most by frugal and responsible consumers, whereas the comfort, environmental friendliness, and longevity of MSWB are important to consumers who identify themselves as ‘*thoughtful spenders*.’ The study suggests that both environmental and hedonic young consumers already familiar with the use of wood in housing contribute to a successful bioeconomy in the urban context.

## Introduction

Because of increasing urbanization, the demand for sustainable urban homes is on the rise. The European Union’s updated Bioeconomy Strategy (2018) labels wood materials as a measure to reduce carbon emissions in the construction sector, thereby enabling the transition towards sustainable bioeconomy. The EU’s aim towards a bio-based circular economy promotes objectives like building development with low land-use, and the use of recyclable, innovative, and sustainable technologies. Because timber is a sustainable building material showing promise for innovative technical applications in building construction (see e.g., Ramage et al. 2017; Pelli and Lähtinen 2020; Toppinen et al. 2019), it is re-claiming popularity among policymakers and within the construction sector (Sposito and Scalisi 2019). Despite this re-awakening among industry and civil society stakeholders, the consumer-driven approach that builds on the consumer perceptions and experiences is missing from this discussion (Toppinen et al. 2018).

In Finland, multi-storey wooden buildings (MSWB) were identified as the most evident business opportunity for a sustainable bioeconomy transition (Bosman and Rotmans 2016). Research suggests that increasing the use of wood in the construction sector has environmental benefits, as wood is a lightweight, renewable material (Gustavsson et al. 2010; Høibø et al. 2015). By using wood instead of concrete or steel, the overall fossil-fuel footprint of building construction can be lowered via material substitution (Milaj et al. 2017), although the extent of this substitutions remains unclear (Hurmekoski et al. 2020). According to some estimates, substituting concrete with wood could reduce the energy consumed by construction processes by 40%, while greenhouse gas emissions could be reduced by 35% (Herczeg et al. 2014). Additionally, MSWB also sequester carbon for several decades (Mahapatra and Gustavsson 2008), and therefore support a sustainable forest-based bioeconomy transition more than other short-lived timber products, like bioenergy or paper (Näyhä 2019). Amidst these positive possibilities, policy support was ultimately instrumental in the diffusion of MSWB in Finland (Vihemäki et al. 2019). The resulting policy-push has contributed to an overall increase in the volume of wood-based material used in residential construction. Annually, MSWB account for 5% of the apartments in multi-storey buildings (Ibid.).

Despite the success of policymakers in supporting MSWB diffusion, the cultural acceptance for MSWB–including whether MSWB are considered sustainable (Vainio et al. 2019)–has yet to be determined. Moreover, while researching the consumers’ environmental considerations, it should be considered that they intertwine with the social, cultural, economic, and psychological aspects of consumption (Wilk 2002). Environmentally speaking, research on sustainable housing is usually linked to issues of energy consumption within buildings and households (e.g., Gram‐Hanssen 2011; Luo et al. 2017), rather than the ecological impacts of differing building materials (e.g., Hildebrandt et al. 2017). Culturally speaking, the house as a home signifies more than a functional shelter: a house is also a symbol of social relations, and the building and the furniture in itself are important to people’s connection to their home (Gram‐Hanssen and Bech‐Danielsen 2004). Thus, research on consumer perceptions regarding housing is needed to bridge these multidimensional aspects.

Understanding people’s expectations and experiences is vital for consumer-driven business strategies to succeed in the bioeconomy (Carù and Cova 2007; Toppinen et al. 2018). Yet, the inclusion of the human dimension, such as resident perceptions and experiences, is often missing from housing and construction-related issues (Gram‐Hanssen 2014). This is especially interesting given that user-centric service innovations are considered focal to the development of sustainable societies (Calabrese et al. 2018). Such arguments favoring the inclusion of a social dimensions run parallel to the framework of socio-technical transitions, where a systemic change towards sustainability requires the inclusion and understanding of multiple actors, including consumers (e.g., Geels 2002; McMeekin and Southerton 2012; Geels et al. 2015). However, there is limited knowledge about how consumers perceive the use of wood in multi-storey buildings. Previous research mainly focuses on investigating the consumer’s overall perceptions of wood as a building material (e.g., Gold and Rubik 2009; Høibø et al. 2015; Larasatie et al. 2018; Luo et al. 2018; Lähtinen et al. 2019; Viholainen et al. 2020), but research is needed on how these perceptions about building materials differ among young consumers.

The literature suggests that young consumers’ housing choices depend strongly on price, location, and social status (e.g., Hoolachan et al. 2017; McKee et al. 2017), rather than building material preferences. Research suggests that young people would have a greater interest towards the use of wood in housing if wooden homes were a more affordable option (Roos and Hugosson 2008; Hakala et al. 2015; Høibø et al. 2015). This signals a tight relationship between the young consumers’ finances and their capacity to act out on their environmental preferences. Indeed, consumer research shows that the consumption styles of young people vary in terms of financial and environmental aspects (e.g., Autio and Wilska 2005; Autio et al. 2009). However, earlier studies focused on young consumer perceptions in contexts other than housing (e.g., Wilska 2003; Wilska and Pedrozo 2007). Thus, exploring the differences between students’ consumption styles in housing deserves more research attention for two major reasons. Firstly, many students are young adults with a high probability of becoming future homebuyers. Secondly, they have entered the housing in an urban environment with varying housing experiences.

Previous studies have separately examined either young people's views on housing materials or young people’s self-perceived consumption styles. Our study aims to enrich the literature and fill a knowledge gap in sustainable housing research by exploring the relationship between ‘consumption styles’ and perceptions about using wood as a construction material. The aim of this paper is to understand differences in students’ perceptions towards MSWB and to show how young urban consumers perceive this unique sustainability-driven building solution. To explore the linkages between consumption style and students' perceptions of the use of wood in multi-storey buildings, we analyze empirical survey data. The survey asks students living in the Helsinki metropolitan area how they perceive MSWB, what kind of previous knowledge and experiences they have about living in wooden homes, and how they self-identify their ‘consumption style.’ In the next section, we review existing studies about consumer perceptions of wood as a building material, and studies on financial and environmental responsibility as consumption styles. Based on the results of these studies, we suggest five propositions^1^ for the analysis: two of the propositions stem from the research stream of consumer perceptions of wood as a building material and three are based on the previous studies on consumption styles.

## Theoretical dimensions

### Consumer perceptions of wood as a building material

The literature on consumer perceptions of wood as a building material is evolving. Wood manifests both positive and negative perceptions among consumers. The literature suggests that “soft” factors, such as aesthetics, wellbeing, and environmental friendliness, are highly valued features of timber frame houses; they are given a high degree of importance among German consumers (Gold and Rubik 2009). Other studies also document consumers valuing wood as building material, especially features like aesthetic beauty and comfortable living (e.g., Larasatie et al. 2018). The aesthetics of wooden interior are appreciated by young people, but they also perceive wood as expensive and question whether such wood products are environmentally sustainable (Roos and Hugosson 2008; Hakala et al. 2015).

On the other hand, a survey study from Norway eliciting building material preference shows that urban consumers are skeptical about the physical durability of wooden buildings (Høibø et al. 2015). Similarly, respondents from Gold and Rubik’s (2009) study also have doubts about the stability of wood as a building material. Furthermore, Gold and Rubik’s respondents were skeptical of other aspects including fire resistance, maintenance ease, longevity, modernity, and the cost and value of construction with wood. Because of the many background variables that play a role in determining preferences, one cannot assume that consumers have homogenous preferences towards housing. In the Finnish context, Lähtinen et al. (2019) argued that consumers who appreciate the ecological and technological benefits of wood differ from those consumers who value the aesthetic and wellbeing benefits of wood.

The environmental sustainability of a bioeconomy—including the use of wood in construction—is not uncritically accepted by consumers and citizens (Vainio et al. 2019). For example, Larasatie et al. (2018) who mapped the beliefs of urban, North-American consumers towards tall wood buildings (i.e., MSWB more than five stories), reported that consumers identified tall wooden buildings as being than buildings made from concrete and steel, but were concerned that tall wooden buildings were a cause of deforestation. Moreover, in previous studies, the environmental aspects of consumer perceptions were connected to both aesthetic (Gold and Rubik 2009) and technical (Lähtinen et al. 2019) properties of wood—thus, there is no consensus on whether environmental sustainability connects with the “hard” technical or “soft” aesthetic values associated with housing.

It is evident that consumer knowledge, experience, and lifestyles all play an important role in material preferences (Ærø 2006). For example, Høibø et al. (2015) notice a positive association between low levels of knowledge and the understanding of wood durability—they found that childhood experiences living inside wooden homes shape attitudes towards wood in urban housing. Similarly, Larasatie et al. (2018) found that there is a low level of knowledge about MSWB, and that a respondent’s level of knowledge about wood buildings affects how the respondent views wood buildings. Overall, concerns related to fire safety or earthquake resistance were common, however, those previously familiar with tall wood buildings were less prone to consider them susceptible to fire or to consider their maintenance as labor-intensive. Based on the literature, the first two propositions suggest that there are differences in overall perceptions about MSWB, and that familiarity (i.e., knowledge and experiences) plays an important role in shaping these perceptions.

#### **Proposition 1**

*Students’ perceptions about the use of wood in MSWB will differ in terms of technical and aesthetic factors*.

#### **Proposition 2**

*Students more familiar with wooden houses are more positive about MSWB*.

### Financial and environmental responsibility in consumption styles

The meaning of style is an integral part of modern youth culture (Croghan et al. 2006). Usually, style is expressed by consuming music, fashion, or food. One’s relationship with money, and to spending money, is a source of identity as well. We apply the concept of *consumption styles*—the understanding of oneself as a consumer (Autio et al. 2009)—to categorize consumers. In our approach, ‘consumption style’ stems from the research tradition of consumer self-understanding, which identifies typologies like green consumers, hedonistic consumers, spenders, and financially sensible consumers (Wilska 2003; Autio 2004; Brusdal and Lavik 2005; Autio et al. 2009; Giesler and Veresiu 2014). Both consumers’ relationship with money and consumers’ environmental attitudes are identified as integral dimensions of young consumers’ understanding of themselves as consumers (Autio et al. 2009; Autio and Wilska 2005; Perera et al. 2018). Previous consumer research recognizes that the consumption styles of young people can play an important role in the individual’s capacity to enact their consumer behaviors. For example, consumption styles are linked to an individual’s capability to uptake novel technologies (e.g., Wilska 2003; Wilska and Pedrozo, 2007; Collins 2019), to consumer resilience (Ponsford 2011), and to the use of small instant loans (Autio et al. 2009). Because our focus is on perceptions and appreciations of wood as a building material overall, we adopt the broad understanding of consumption style, similar to McCarty et al. (2007), instead of the traditional focus on consumer decision-making styles (Sproles and Kendall 1986; Akturan et al. 2011; Maggioni et al. 2019). Using the concept of consumption style, we interlink the environmental aspect of consumption to responsibility of consumption and individual’s relationship with money.

Classifying consumption styles allows us to connect economic dimensions to ecological orientations. Previous research argues that the economically limited situation of young consumers restricts them from purchasing ecological wooden furniture (Hakala et al. 2015). Furthermore, studies on consumers’ willingness to pay a price premium for sustainability labels show that although consumers recognize and assign importance to the sustainability aspects of products, it may not contribute to their willingness to pay a price premium (Shao and Ünal 2019). Luo et al. (2018), who studied price premium acceptance for modern wooden structure residences in Japan and China, found price premium acceptance to be higher in Japan due to affective factors. However, there is no prior research studying willingness to pay premium for wooden homes in connection with either consumption styles or consumers’ relationship with money. Based on the literature above, we formulate three propositions to be studied.

#### **Proposition 3**

*Consumers with environmental consumption style are more positive towards MSWB than other consumer types*.

#### **Proposition 4**

*Consumers with hedonistic consumption style appreciate aesthetics of wood more than other consumer types*.

#### **Proposition 5**

*Consumers with a loose relationship to money are more likely willing to pay a price premium for MSWB compared to others consumer types*.

### Measures

Based on the literature above, we theorize that the key concepts affecting the student’s perceptions of MSWB are (1) Aesthetic attractiveness of wood, including other “soft” factors like coziness; (2) Technical qualities, including fire safety, air quality, and longevity; (3) Environmental sustainability; and (4) Familiarity, including knowledge about MSWB and exposure to wooden building materials in the childhood home. Furthermore, we use (5) Consumption style as an indication of how the perception of self as a consumer relates with student’s perceptions of MSWB. Thus, these five concepts are at the core of this research. Table 1 indicates how each concept is operationalized.

**Table 1:** Operationalized statements in the questionnaire based on key literature

Statements	Source
*Perceived aesthetics*	Gold and Rubik (2009)
After seeing the pictures, my first impression of the facades of the buildings is positive	
The appearance of building is pleasant	
I would like to live in such an apartment building	
Houses built from wood are warm and cozy	
The MSWB seems more comfortable to live in in comparison to a concrete apartment building	
Wood suits better to vacation homes than to urban context	
*Perceived technical qualities*	Gold and Rubik (2009), Høibø et al. (2015), Lähtinen et al. (2019)
The indoor air quality is better in the MSWB compared to concrete apartment buildings	
I believe the MSWB to last in use (longevity)	
The fire safety of MSWB is a challenge to secure	
*Perceived environmental sustainability*	Høibø et al. (2015), Larasatie et al. (2018)
Houses built from wood are ecological (for instance, wood is a carbon sink)	
*Familiarity*	Høibø et al. (2015), Larasatie et al (2018)
I have never heard of wooden apartment buildings	
I have heard talk or read a newspaper	
I am interested in the subject and I know something about it	
Wood block construction is familiar to me via studies/work	
I have lived in or visited a wooden apartment block 2000s	
What material was used in the structures of your childhood home (before age 16)?	
*Consumption style*	Wilska (2003), Autio et al. (2009)
I perceive myself as a sensible consumer—‘I hardly ever buy anything unnecessary’	
Environmental issues (including recycling, environmentally friendly products) are an important part of my consumption and spending of money	
I am unable to handle money: ‘as soon as it came, it went	
I enjoy spending money on shopping, restaurants, or other leisure consumption	
*Willingness to pay*	Luo et al. (2018)
I would be willing to pay higher rent for an apartment in a wooden apartment compared to a similar apartment in a concrete apartment building	

Statements are measured on five-point Likert scale (1 = fully disagree–5 = fully agree)

**Fig. 1:**
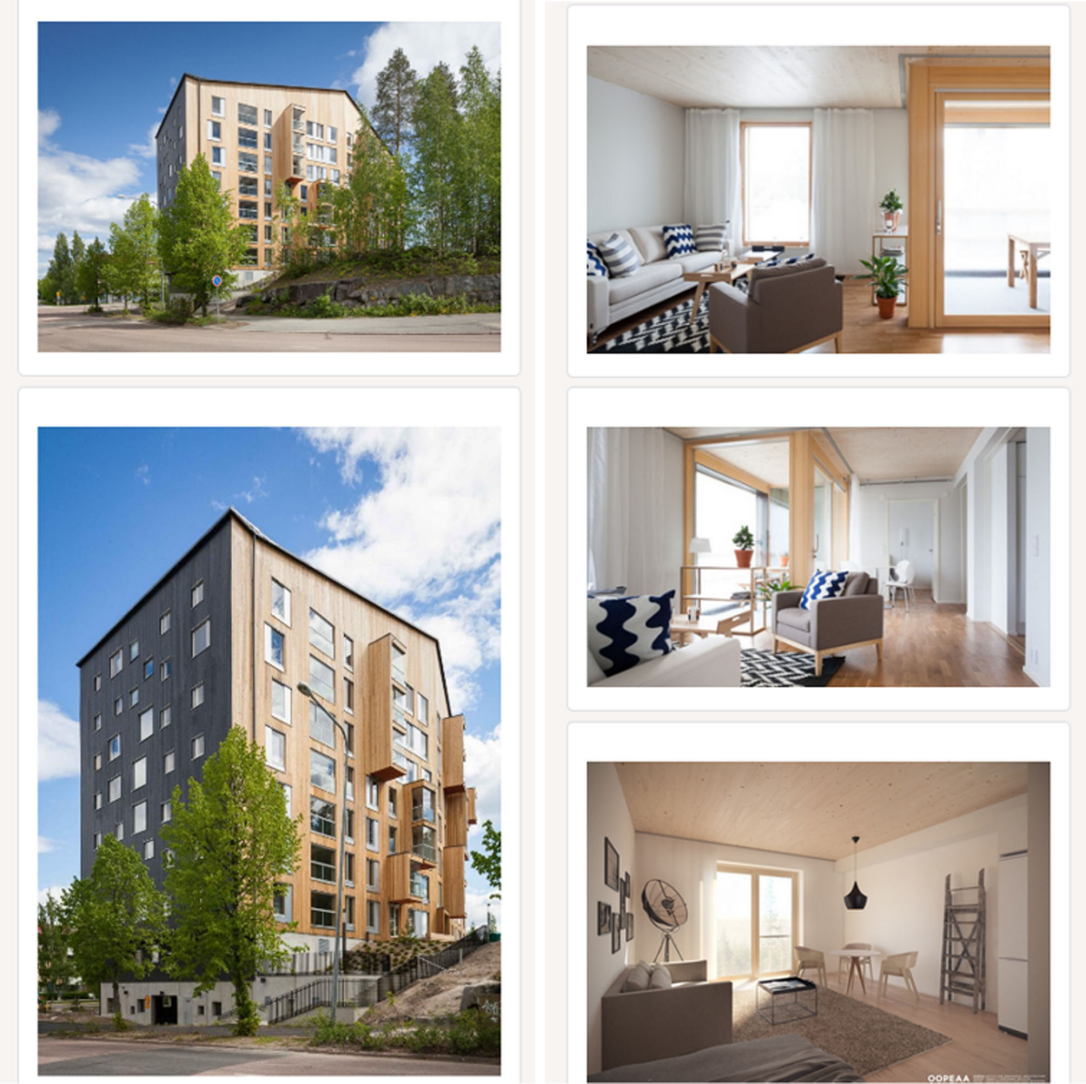
Pictures of a wooden multi-storey student apartment buildings shown to respondents (*Source* OOPEAA, Jukka Auerniitty)

When mapping respondents’ perceptions about the *aesthetics of wooden buildings*, we decided to use pictures of the interior and outdoor architecture of a wooden student apartment building located in Central Finland as a stimulus (see Fig. 1). After seeing the pictures, respondents were asked about the “soft” dimensions of MSWB in line with Gold and Rubik (2009). These survey items were designed to induce the first reflections of the respondent based on the images seen.

The survey items measuring the perceived *technical qualities of MSWB* follow the items used in previous studies measuring perceptions about durability, longevity, and fire resistance of MSWB (Gold and Rubik 2009; Høibø et al. 2015). We also included an item concerning indoor air quality, following Lähtinen et al.’s (2019) finding that it was a relevant technical property of wood among citizens.

In the literature, the sustainability and environmental properties of MSWB are considered as both “soft” (i.e., combined with aesthetics of wood (Gold and Rubik 2009)), and “hard” (i.e., combined with the technical properties of wood (Lähtinen et al. 2019)) factors. In this study, *environmental sustainability* was treated as a separate concept than either *technical qualities* or *aesthetics*. Perceptions about *environmental sustainability* were operationalized through a singular statement (i.e., Houses built from wood are ecological (for instance, wood is a carbon sink)). This decision supplemented our aim to explore the link between consumption style and environmental issues.

Items measuring *familiarity* are based on Larasatie et al.’s (2018) argument of knowledge as a key component of MSWB acceptance. As previous studies have shown, familiarity also connects to background factors, such as the childhood homes building material (Høibø et al. 2015; Larasatie et al. 2018). Thus, childhood experience was one key background factor that was examined.

*Consumption style* is based on Autio et al. (2009) study exploring young consumers’ understanding of themselves as consumers based on the consumers’ relationship to money. The multiple consumption style typologies were operationalized through four varying five-point Likert scale attitude statements. To categorize the different types of responsible consumers, statements recognizing financially sensible consumers and environmentally aware consumers were used. Furthermore, in order to connect consumption styles with consumers’ willingness to pay price premium for wood as a construction material, a supplementary statement in line with Luo et al. (2018) was added.

## Data and analysis

The data consisted of responses provided by student occupants of apartments rented out by HOAS (Helsinki Student Housing Foundation) in the Helsinki metropolitan area (Helsinki, Espoo, and Vantaa) in Finland. The survey was designed and employed using Google Form. It included structured questions assessed through five-point Likert scale measures, and open-ended questions. Prior to data collection, five researchers tested the questionnaire. Based on the pre-testing, the order of the questions was reorganized to improve question flow, and the questionnaire was shortened (e.g., general questions related to housing preferences were omitted). The questionnaire was provided in Finnish. Translation into English was made by the first author with the help of key references (e.g., Autio et al. 2009; Gold and Rubik 2009; Høibø et al. 2015; Larasatie et al. 2018; Lähtinen et al. 2019), and was subsequently cross-checked by the author team.

The basic population of HOAS occupants is approximately 18,000 students. Using gender as the stratification, a stratified random sample of 5000 students was selected from the HOAS email address registry; the invited participants were equal parts of men and women. The questionnaire was sent to respondents via email. Each email included an information letter about the study, and a webpage URL-address to the survey. One reminder email was sent. 531 students responded to the survey (i.e., response rate 10.6%). Today, young people prefer rapid communication channels, such as Instagram, Twitter, and WhatsApp (e.g., Fardouly et al. 2018; Bano et al. 2019), therefore sending a questionnaire by email does not reach young people at the same rate as rapid communication. While the response rate reflects a low proportion of the initial sample, the explorative nature of the study makes the response rate acceptable. Additionally, the low rate of missing values and unfinished questionnaires resulted in a large number of high-quality answers. Furthermore, many respondents answered the open-ended questions and we were able to gauge concerns about limited response rates through these open comments. Prior to analysis, the collected Google Form responses were transformed via Excel into a SPSS readable form.

Analyses were conducted using SPSS (Ver.22) statistical software. First, we screened responses for normality and distributional properties. Second, we reviewed the open-ended responses to evaluate the coherency of the respondent’s behavior in reference to their answers on five-point Likert scale statements (e.g., missing values and non-response error). As 80% of the respondents answered the open-ended question *‘Describe wood as a building material*,’ we could deepen our view of individual responses and corroborate the associated verbal responses. The high response rate to the open-ended question indicates that students were committed to answering the survey and it thus reinforces the reliability of the study.

Multivariate statistical analyses were conducted on the scales measuring key dimensions (i.e., perceptions of MSWB, perceptions of oneself as a consumer, and perceptions of willingness to pay). Common correlations, factor analytic (FA) stability of the dimensionality, and ANOVA testing of the resulting differences were applied. Cluster analysis was used to divide respondents into consumption style typologies.

Our analysis has three major limitations. First, results from a student population living in one area of a single country cannot be generalized. Second, the overall low survey response rate of 10.6% coupled with the high number of ‘*don’t know*’ responses may affect the conclusiveness of the study. Third, some items contained a high frequency of ‘*don’t know*’ responses and may lack reliability. On the one hand, items with a high frequency of ‘*don’t know*’ responses may reflect poor question development, as not all key terminology in the survey was unidimensional. For example, the concept “longevity” may evoke either technological or environmental considerations. Such double-loaded items can result in response ambiguity and preference towards the ‘*don’t know*’ response. On the one hand, the high number of *‘don’t know*’ responses may also indicate a lack of knowledge about the subject. This is corroborated by the high frequency of students responding that they had not previously heard about MSWB (23%). Because students primarily had challenges answering complex questions—like whether wood acts as a carbon sink (item 8)—it seems reasonable to assume that ‘*don’t know*’ responses indicate a lack of knowledge about the subject rather than poor question development. In addition, previous research indicates there is an inability to respond to complex questions about forest bioeconomy concepts is associated with the lay-person’s lack of knowledge about the subject (e.g., Vainio et al. 2019).

## RESULTS

### Descriptive statistics

As Table 2 describes, respondent age ranged from 18 to 61 years, with the median being 25 years. 59% of respondents were female and 41% were male. 42% of the students lived alone, 40% lived with their spouse, and the rest had other types of accommodation arrangements. Only 6% of respondents had children. Most respondents (42%) originated from the metropolitan area, 28% were from a medium-sized city, and 17% had a rural background. The smallest group (13%) originated from ‘a big city’ different than the metropolitan area. In Finland, this means cities with more than 100 000 inhabitants. The small number of respondents from the ‘big city’ group may be explained by those locations having their own educational institution similar to those found in the metropolitan area.

**Table 2:** Background questions and description of the sample

*Variable*	*n*	%
*Gender*		
Female	309	58.4
Male	220	41.6
No answer	2	0.4
*Age (year of birth)*		
1962–1985	24	4.5
1986–1990	72	13.6
1991–1993	125	23.5
1994–1996	197	37.1
1997–2001	98	18.5
*Current educational institution*		
Vocational educational institution	33	6.2
University of applied sciences	162	30.6
University	294	55.6
Other	40	7.6
*Most-part of life home location (urban–rural)*		
Metropolitan area	225	42.4
Large city (e.g., Tampere, Turku, Oulu, Kuopio)	70	13.2
Small and/or medium-sized city,	148	27.9
Rural area or countryside	88	16.6
*Childhood home building material*		
Wood	100	18.9
Wood and change	175	33.0
Change (e.g., brick/concrete/steel)	248	46.8
I don't know	7	1.3
*Childhood home building type*		
Detached house	187	35.2
Row house	30	5.6
Semi-detached house	12	2.2
Multi-storey apartment building	81	15.2
*Knowledge on wooden apartment buildings*		
I have never heard of wooden apartment buildings	123	23.2
I have heard talk or read a newspaper	271	51.0
I am interested in the subject and I know something about it	90	16.9
Wood block construction is familiar to me via studies/work	24	4.5
I have lived in or visited a wooden apartment block 2000s	23	4.3

To check for non-response bias, we compared three background variables (i.e., age, type of educational institution, and gender) of the total population group versus those of the survey respondents’ group. We found that the median age of the total population was 24 while in the survey respondent group it was 25 (see: Table 2). The difference is non-significant, as 95% of the students in the total population group are between 18 and 35 years of age. The type of educational institution displayed a clear difference between the amount of student studying in the universities (56% in the data vs. 46.8% in the basic population). Thus, the responses are slightly biased towards university student views. In the survey respondent group, gender was also inclined towards female respondents. In the basic population, the 51.4% are female and 48.1% are male, the rest identify as “other.” However, the survey was 58.8% female and 40.8% male, the rest being “other.” Thus, gender is the background variable resulting in the most bias.

Of those students originating from the metropolitan area, most (60%) had previously lived in buildings made from a material other than wood. Of the students originating from medium-sized towns and rural areas, most (66%) had previously lived in buildings made either of ‘*wood’* or ‘*wood and other*’ materials. The childhood homes of the latter group were mainly single-family houses (60%), compared to the metropolitan area respondents, of which only a minor portion (18%) had lived in a single-family home.

Regarding the background question about the childhood home construction material, most respondents answered with ‘*brick, concrete or other similar materials.’* Note that it is possible those who lived in a brick clad house perceive the house as being made of bricks even though the structure might have been wooden (e.g., approximately 80% of detached single-family houses in Finland are constructed with wooden load-bearing structures (Hurmekoski et al. 2015)). The second most frequent structural material experience was with wood. To facilitate cross-tabulation between individuals with any previous experience living in a wooden home versus those who had no experience living in a wooden home, we created a variable that combined the responses ‘*wood’* and ‘*wood and other*’ into one group, while ‘*brick, concrete, or other similar materials’* were left as a second group. ‘*I don't know’* responses were omitted from testing. This allowed a clearer breakdown between those who had any experiences living in a wooden home and those without any experience.

### Students’ perceptions of MSWB

To gauge the dimensionality of the MSWB perceptions, we computed several factor models from 10 items using principal axis factoring with varimax rotation. As we are dealing with exploratory methods, the factor solutions are arbitrary in that no “correct” number of factor dimensions can be concluded to exist. The recommendation is to run parallel competing models (e.g., Bagozzi and Edwards 1998; Hair et al. 2009) and choose the model that is closest to the structure of the theory-proposed model. Thus, after gauging the stability of the solution by varying the number of factors in the matrix between two-, three- and four-, a three-factor solution was chosen to be most representative. Compared to the two-factor and four-factor solutions, the two-factor solution produced a good level of explanatory power (*R*^2^ = 49.8%) and a logical loading structure between interrelated items (see Table 3).

**Table 3:** Three factors describing dimensionality of the student MSWB perceptions and ‘*don’t know*’ answers

	Number of ‘*do not know*’ responses	Factor label		
		Perceived comfort, environmental friendliness, and solidity	Perceived aesthetic attractiveness	Perceived technological and stylish dubiousness (R)
After seeing photos of MSWB exterior				
1. After seeing the pictures, my first impression of the facades of the buildings is positive	–		0.730	
2. The appearance of building is pleasant	–		0.922	
3. I would like to live in such an apartment building	11		0.559	
After seeing photos of MSWB interior				
4. Houses built from wood are warm and cozy	5	0.716		
5. The MSWB seems more comfortable to live in in comparison to a concrete apartment building	89	0.751		
6. The indoor air quality is better in the MSWB compared to concrete apartment buildings	209	0.666		
7. I believe the MSWB to last in use (longevity)	85	0.531		0.435
8. Houses built from wood are ecological (for instance, wood is a carbon sink)	107	0.628		
9. (Reverse) The fire safety of MSWB is a challenge to secure	167			0.585
10. (Reverse) Wood suits better to vacation homes than to urban context	58			0.572
Cronbach’s alpha		0.807	0.775	0.504
R2 = 49.8%				

We further tested the stability of the solution by computing unidimensional factors for each sequence of questions (i.e., items 1 to 3; 4 to 8; and 9 to 10—see Table 3). These three separate unidimensional solutions were compared to the composite FA of the whole series of questions. The results showed that the unidimensional factors correlated over 0.95 with the three-dimensional simultaneous solution. Thus, using the three-dimensional solution to describe the perceptions of the students towards MSWB seems appropriate. Due to high number of ‘*do not know*’ responses in items 5 to 10 (see Table 3), a sensitivity analysis was conducted by creating two alternative factor solution models. In one model, ‘*do not know*’ responses were converted into missing values. In the second model, ‘do not know’ responses were converted into neutral values representing the middle of the scale. The results of both these analyses produced similar outcomes. The clearest difference between the two models was that the model replacing ‘*do not know*’ with a middle scale response produced slightly lower values of variance accounted for by the factors (R2) and lower Cronbach’s alpha figures (see Table 3). As a precaution, we resorted to using the middle of the scale alternative, as it captures the degree by which the students’ perceptions of the dimensions are weak or non-existent.

Table 3 reports the final three-dimensional solution. The Cronbach’s test figures support the observation that the solution appropriately captures the variance in the data and suggest a good level of reliability in the two first factors, with the third factor being 0.5. We labeled the first factor ‘*Perceived comfort, environmentally friendliness and solidity*,’ as the factor grouped items attributing wood to perceived wellbeing, durability, and environmental benefits. The second factor grouped elements of visual appeal, such as pleasant looks, and was thus named, ‘*Perceived aesthetic attractiveness*.’ Unlike the first two factors, the third factor contained attributes reflecting suspicion towards using wood in buildings. As such, it was labeled, ‘*Perceived technological and stylish dubiousness.’*

Notably, the item ‘longevity’ (statement 7) loaded into two factors: Factor 1 ‘*Perceived comfort, environmental friendliness and solidity,’* and Factor 3 ‘*Perceived technological and stylish dubiousness*.’ Despite the double-loading, the item was left in the FA. This is justified because the item is a key measure of the scale itself. In future studies, the statements wording should be altered, because in the present form, the question taps into two key dimensions of the model instead of just one. Leaving the item in the FA is also justified because principal axis factoring is an orthogonal approach, therefore the acquired factors are likely correlated since cross-loading indicates an association between two factors.

### Appreciation of wood in relation to consumption style

Respondents’ self-reported their consumer identity through a series of four statements (see: Table 3). The four statements produce a two-dimensional factor solution that describes respondent orientations towards their spending and responsibility of consumption. We named these two factors (i.e., dimensions of consumption styles): ‘*Money spending and hedonism*’ and ‘*Financially and ecologically responsible consumption*’ (Table 4).

**Table 4:** The dimensions of consumption styles based on the understanding of oneself as a consumer

	Money spending and hedonism	Financially and ecologically responsible consumption
I perceive myself as a sensible consumer—‘I hardly ever buy anything unnecessary.’ (financially sensible consumers)		0.630
Environmental issues (including recycling, environmentally friendly products) are an important part of my consumption and spending of money. (ecological consumers)		0.593
I am unable to handle money: ‘as soon as it came, it went.(spenders)	0.522	
I enjoy spending money on shopping, restaurants, or other leisure consumption. (hedonists)	0.690	
*R*^2^ = 42%, Cronbach =	0.54	0.56

**Fig. 2:**
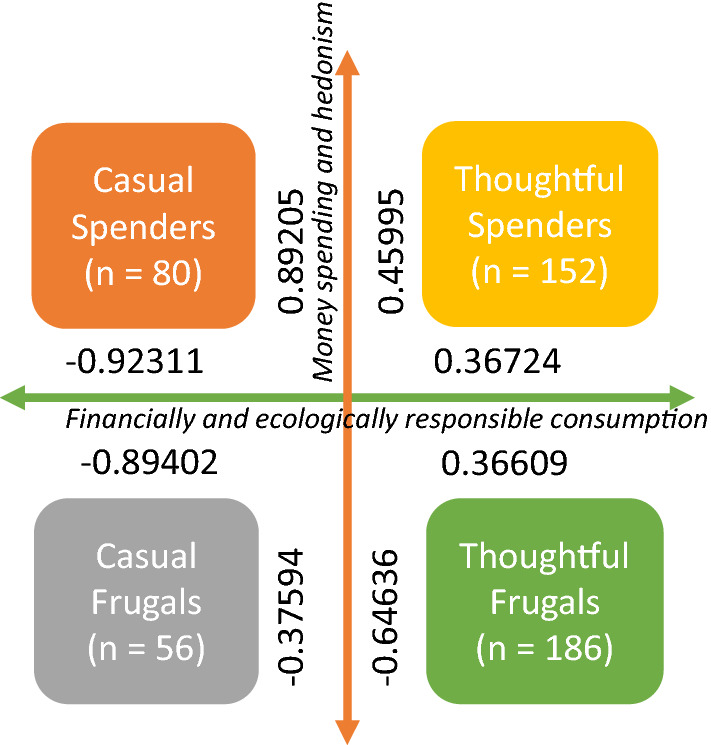
Clustered consumption styles described on the (high/low) dimensions of ‘Money spending and hedonism’ and ‘Financially and ecologically responsible consumption’

Because respondent could exist as high or low on the two dimensions, we examined if any distinct groups formed based on the consumption style dimensions of each respondent. The two-dimensional FA model of the respondent consumption style (Table 4) was subject to a k-mean cluster analysis to determine respondent groups with similar orientations to ‘*Money spending and hedonism*’ and ‘*Financially and ecologically responsible consumption*.’ That is, we used the factor scores of each respondent on the two-dimensional factor solution to compute the cluster membership. The analysis generated four cluster centers. We named the four clusters (i.e., consumption styles): ‘*Casual frugals,’* ‘*Casual spenders*,’ ‘*Thoughtful frugals*,’ and ‘*Thoughtful spenders*’ (see: Fig. 2).

Figure 2 depicts the four consumptions styles according to how high or low a respondent aligns to the two dimensions (i.e., ‘*Money spending and hedonism*’ and ‘*Financially and ecologically* *responsible consumption*’). ‘*Casual frugals*’ are low on both dimensions, ‘*Thoughtful spenders*’ are high on both dimensions, and ‘*Casual spenders*’ and ‘*Thoughtful frugals*’ are the opposed combinations of high and low.

We used ANOVA to determine how the four consumptions styles differed from each other. We found that ‘*Casual spenders*’ were ready to favor living in MSWB in comparison to multi-storey concrete buildings, if they cost the same to rent or buy. However, this difference was only significant compared to ‘*Thoughtful frugals*’ (*p* = 0.066). When analyzing the four consumption styles in relation to willingness to pay more for renting or buying an apartment in a MSWB, the differences were more considerable. Both ‘*Thoughtful spenders*’ (*p* = 0.046) and ‘*Casual frugals*’ (*p* = 0.028) were more willing to pay a premium for living in a MSWB than the two other groups. But it is important to note that while ‘*Thoughtful frugals*’ showed willingness to pay a premium against ‘*Casual spenders’*, these results were not statistically significant.

**Fig. 3:**
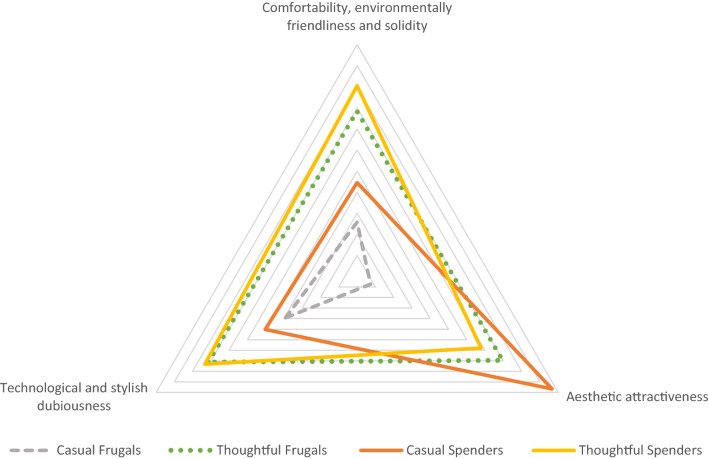
Consumption styles and their relationship to MSWB perceptions

Using factor score variables, we computed the association between consumption clusters (Fig. 2) and MSWB perceptions (Table 2). The results depict that consumption styles have a significant association with how MSWB are perceived among respondents. All four consumption style groups possess radically different perceptions about MSWB, except ‘*Thoughtful spenders’* and ‘*Thoughtful frugals,’* both of which have similar outlooks towards MSWB (see: Fig. 3). The visually identifiable differences in Fig. 3 are also statistically significant, varying from the levels from 0.002 to 0.073.

Lastly, we analyzed how the level of the knowledge about MSWB and exposure to living in wooden homes during childhood (i.e., childhood home building material) affects perceptions towards MSWB. We measured the relationship between the level of knowledge about MWSB and perceptions towards MSWB through two approaches: a self-reported scale (see: Table 1), and as a reflection in the amount of ‘*do not know*’ responses obtained in the survey (see: Table 2). On the self-reported scale, respondents answer whether they ‘*had not heard about MSWB*’ (*n* = 113), ‘*had heard or read about it*’ (*n* = 263), or ‘*had some interest in the subject*’ (*n* = 87). Those familiar with MSWB through their studies (*n* = 22), and those with previous experiences living in MSWB (*n* = 22) constitute the minority of respondents. The responses measuring student knowledge about MSWB follow an ascending sequence (i.e., ‘*had not heard about MSWB*,’ ‘*had heard or read about it*,’ ‘*had some interest in the subject*’). Those with knowledge ‘*heard or read in the newspaper*’ are less likely to ‘Perceive comfort, environmentally friendliness and solidity’ in MSWB. The ‘*Perceived aesthetic attractiveness*’ factor did not show a relationship to any knowledge groups. Notably, the ‘*Perceived technological and stylish dubiousness*’ group was made up of a significantly higher number of respondents who answered they had ‘*never heard*’ of MSWB (*p* = 0.005 to 0.000).

The building material used in the respondents childhood home affected the respondent’s perceptions of MSWB. Those with exposure to wood earlier in life were significantly (*p* = 0.025 to 0.009) less suspicious towards wood as a building material (Table 2, Factor 3) compared to those with a history of living in houses made from ‘*other materials*.’ Childhood experiences living in a wooden home did not have a relationship to the ‘*Perceived comfort, environmentally friendliness and solidity*’ of wood (Factor 1) or the ‘*Perceived aesthetic attractiveness*’ of wood (Factor 2).

## Discussion

In this section, we will discuss the results in relation to our five propositions and existing literature, the validity of our study, and the relevance of the results for the bioeconomy transition. Firstly, the results indicate that proposition 1 (Perceptions of students on the use of wood in MSWB differ in terms of technical and aesthetic factors) is supported, as students either appreciate MSWB for its *aesthetic qualities* or for qualities related to *wellbeing, environment, and longevity*, including technical factors. However, in our model aesthetics stood out own its own, whereas technical aspects connected with other “soft” factors, namely wellbeing and environment. This differs from Lähtinen et al.’s (2019) study showing consumers appreciated either the wellbeing and aesthetic qualities of MSWB or the technological and environmental qualities of MSWB. Moreover, in our study, there were contradicting perceptions about the technological properties of wood used in MSWB. Even though results showed that students had positive attitudes towards the longevity of wood, they also held suspicions about these technological properties. This is a similar finding to other studies (Gold and Rubik 2009; Høibø et al. 2015; Larasatie et al. 2018).

Also, as the second proposition (2: Those students more familiar with wooden houses are more positive about wood in apartment buildings) suggested, the results showed that either experiences living in a wooden house or having interest towards MSWB result in more positive perceptions of MSWB. Thus, a higher level of knowledge and childhood exposure to wood construction materials protect the respondents from suspicious attitudes towards the usage of timber in MSWB (see also: Høibø et al. 2015; Larasatie et al. 2018). In addition to corroborating these findings, we found that respondents with childhood exposure to wood were more likely to be ‘*Thoughtful consumers*’ (i.e., consumers with higher financial and ecological responsibility in their consumption style).

To study the connections between consumption styles and perceptions of the use wood in MSWB, this study parsed consumption styles into two dimensions: ‘*Financially and ecologically responsible consumption*’ (i.e., consumers with both financial and ecological responsibility) and ‘*Money spending and hedonism*’ (i.e., consumers with both readiness to use money and hedonistic consumption attitude). While self-understanding as a consumer has been analyzed in previous studies (e.g., Wilska 2003; Brusdal and Lavik 2005; Autio et al. 2009), bringing the financial and ecological dimensions together in this study is a novel approach that revealed differences between consumption styles and MSWB perceptions. The results indicate that yes, the view on oneself as a consumer relates to how the individual perceives MSWB. Firstly, consumer’s environmental orientation associates with both positive and negative perceptions regarding MSWB, therefore the third proposition (Proposition 3: Consumers with environmental consumption style are more positive towards MSWB than other consumer types) is not supported. Instead, secondly, it seems that one’s relationship to money does produce differences in perceptions towards MSWB—spenders appreciate the aesthetics of wood, especially if they are also oriented in responsible consumption (Proposition 4: Consumers with hedonistic consumption style appreciate aesthetics of wood more than other consumer types). For example, the aesthetics of wooden buildings were especially appreciated by ‘*Thoughtful spenders’* who identified themselves as responsible consumers that were ready to use money and held “value for money” thinking. On the other hand, the quality of wood material was especially appreciated by those who were ready to use money but were not very conscious about their consumption, namely the ‘*Casual spenders*.’ Of note is that the willingness to pay a premium for MSWB apartments relates to both the consumers’ hedonic relationship to money and their ecological awareness, as ‘*Thoughtful spenders*’ were more willing to pay a premium for living in a MSWB than other consumer types (Proposition 5: Consumers with a loose relationship to money are more likely willing to pay price premium for wood than others consumer types). This result also adds depth to Luo et al.’s (2018) findings on consumer willingness to pay for wooden homes by identifying the lead consumer profiles of those consumers who are willing to invest in (sustainable) wooden materials.

As explorative studies cannot inherently tackle the validity dimensions directly, it is logical to reflect on the validity of this study. We assume the study is valid given the following justifications. First, the factor analysis model proposed in our study follows theoretical dimensions from preexisting literature (see: Table 1). Second, the final factor analysis model was selected only after being subject to a sensitivity analysis via alternative model testing. This approach is in line with the “competing models” strategy suggested in various methodological references (Bollen 1989; Kline 1998; Maruyama 1998; Hair et al. 2009). Third, the reliability figures obtained from the final model were good (with one exception), thus the final model provides a satisfactory level of confidence about the acquired results. Lastly, we corroborated the final model against the open-ended responses and found the model to be a satisfactory reflection of the phenomena.

The main aim of bioeconomy strategies in the European Union, and in Finland, is to replace fossil-based products with renewable, bio-based materials. Transition towards a sustainable bioeconomy and the development of MSWB does not emerge without both the strategic renewal of companies involved in the building processes and the development of product-service systems to meet the value expectations on the markets (e.g., Pelli and Lähtinen 2020). The results of our study provide new insights in how young people perceive living in MSWB, and how these perceptions are connected to their consumption styles. For the businesses involved in MSWB, a better understanding of the future generations’ housing expectations enhances the possibilities to develop new business models. This, in turn, enables providing new value for consumers through uptake of service innovations. Ultimately, more astute knowledge on differing housing needs among young consumers significantly supports changes in a company’s strategic thinking (e.g., development of consumer-driven business strategies), which are important for the development of a sustainable and bio-based circular economy (see, e.g., Calabrese et al. 2018; Toppinen et al. 2019).

## Conclusions

The aim of this paper was to understand differences in students’ perceptions towards MSWB and to show how young urban consumers perceive this unique sustainability-driven building solution. To conclude, firstly, the study shows that some level of previous exposure to the subject of wood in housing—be it personal experiences or general interest in the topic—removes suspicions related to MSWB. Second, the study suggests that ecological awareness it is not the only underlying value connecting consumers to positive perception of MSWB—the consumers’ approach to spending also connects to the consumers’ perceptions of MSWB. All in all, the results signal that increasing knowledge and awareness of wood in construction, along with the rise of environmentally conscious consumption, is turning the general public’s attention towards issues which are central to the sustainable bioeconomy. As a broad conclusion, we suggest that in a consumer-driven bioeconomy there needs to be better understanding of how to effectively engage differently oriented consumers in more sustainable material choices.

## Footnotes

1 We use the term propositions to describe the expected antecedent–outcome relationship. Ergo, the use of this concept is similar to a hypothesis. Note that we cannot test a hypothesis with the exploratory methods used in this research, therefore the term “proposition” is more appropriate.
